# MASLD prevalence, incidence and global aspects

**DOI:** 10.1007/s00125-026-06726-1

**Published:** 2026-05-21

**Authors:** Sarah H. Wild, Jeffrey V. Lazarus, C. Wendy Spearman, Ponsiano Ocama, Josh Bilson, Xiao-Dong Zhou, Ming-Hua Zheng, Christopher D. Byrne

**Affiliations:** 1 Usher Institute of Population Health Sciences, University of Edinburgh, Edinburgh, UK; 2 City University of New York Graduate School of Public Health and Health Policy (CUNY SPH), New York, NY USA; 3 Barcelona Institute for Global Health (ISGlobal), Barcelona, Spain; 4 Faculty of Medicine and Health Sciences, University of Barcelona, Barcelona, Spain; 5 Division of Hepatology, Department of Medicine, Faculty of Health Sciences, University of Cape Town, Cape Town, South Africa; 6 Makerere University College of Health Sciences, Kampala, Uganda; 7 National Institute for Health and Care Research, Southampton Biomedical Research Centre, University Hospital Southampton and University of Southampton, Southampton, UK; 8 Wenzhou Medical University, Zhejiang, China

**Keywords:** Environment, Global burden of disease, Incidence, Liver disease, MASLD, Metabolic dysfunction, Prevalence, Review, Social determinants of health, Type 2 diabetes

## Abstract

Over the last decade it has become clear that obesity and type 2 diabetes are key to the aetiology, pathogenesis and outcomes of metabolic dysfunction-associated steatotic liver disease (MASLD). The considerable current and potential burden of MASLD on patients and healthcare systems is therefore inextricably linked with today’s global epidemics of obesity and type 2 diabetes. In this review, we discuss estimates of the global MASLD burden in terms of prevalence, incidence, disability from complications and reduced life expectancy associated with the condition. We also highlight the variability in MASLD prevalence estimates among different populations, with a particular focus on the sub-Saharan Africa and Asia Pacific regions, where population increases and the epidemiological transition (a sustained shift in population mortality and disease patterns from infectious diseases towards non-communicable diseases) are occurring most rapidly. Additionally, we consider the impact of social determinants of health, including socioeconomic conditions, structural inequities, care access, food environments and systemic discrimination. We also identify opportunities to advance equity in MASLD prevention, diagnosis and treatment worldwide.

**Graphical Abstract:**

**Figure:**
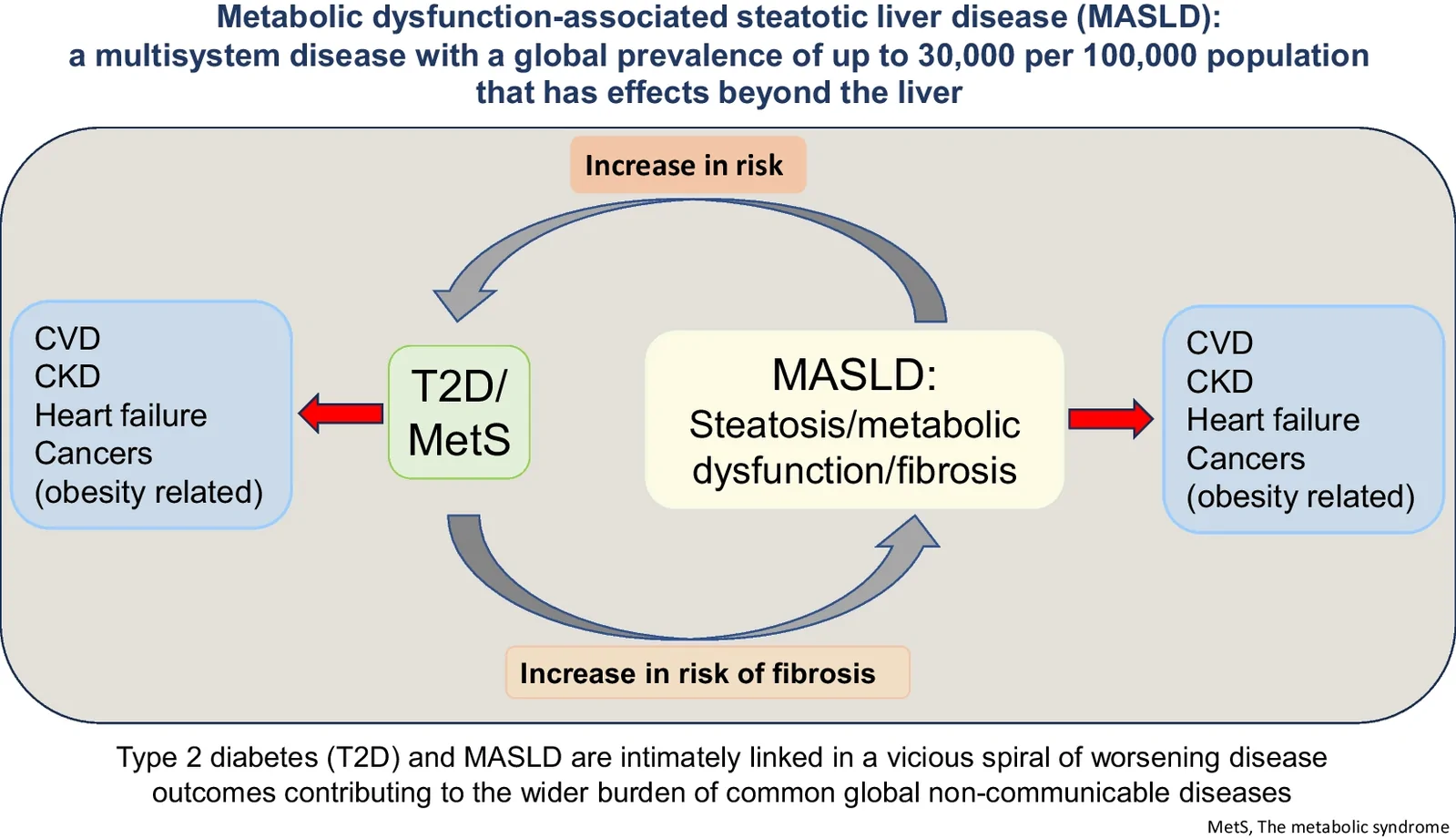

**Supplementary Information:**

The online version contains a slide of the figure for download available at https://doi.org/10.1007/s00125-026-06726-1.

## Introduction

In 2023, non-alcoholic fatty liver disease (NAFLD) was renamed and reclassified as metabolic dysfunction-associated steatotic liver disease (MASLD) [1]. The term MASLD includes the key hepatic complication, metabolic dysfunction-associated steatohepatitis (MASH). As the NAFLD and MASLD definitions are almost equivalent among the general population [2], we use the term MASLD throughout, even when referring to previous studies of NAFLD.

Obesity and associated metabolic syndrome traits, including type 2 diabetes, are strongly associated with MASLD, and MASLD confers an additional risk of CVD independently of conventional CVD risk factors [3]. Time trends in obesity and diabetes prevalence vary between populations and are affected by sociodemographic factors including age, sex, ancestry, ethnicity and socioeconomic status, and also interactions between these factors [4]. In sub-Saharan Africa (SSA), for instance, there is a positive association between socioeconomic status and obesity in low-income countries, with evidence of an inverse association in lower-middle-income countries [5]. This latter pattern is similar to that seen in high-income nations in other parts of the world [4]. Anthropometric data are widely available from population-based surveys, but diabetes screening is rarely performed at the population level, and MASLD data are even less representative as they are largely derived using a variety of diagnostic tests in high-risk populations with high-quality healthcare access. The various sets of global obesity, diabetes and MASLD prevalence estimates rely on the limited available data on diagnosed and undiagnosed outcomes of interest. For example, the proportion of individuals with undiagnosed disease is based on assumptions using data from cross-sectional screening studies that are extrapolated to countries without data. Incidence data are even less frequently available than prevalence estimates and require cohort studies or analysis of electronic health records. As a consequence, all global estimates should be viewed with caution and should be reported in light of the limited data available, potential selection and ascertainment biases and marked heterogeneity within the source data.

MASLD was first identified as a multisystem disease over a decade ago, with effects beyond the liver including higher risks of type 2 diabetes and CVD [6]. MASLD is now known to be associated with an increased risk of a wide range of extrahepatic conditions [7–9]. MASH increases the risk of cirrhosis, end-stage liver disease and hepatocellular carcinoma (HCC) [1], with the risk of adverse liver outcomes in MASLD/MASH primarily determined by the stage of liver fibrosis (e.g. liver stiffness >10 kPa by elastography), severity of metabolic dysfunction (e.g. presence of type 2 diabetes) and other risk factors (e.g. alcohol consumption, genetics, age and sex) [10].

According to the United Nations (UN), the global population is projected to continue growing and will peak at about 10 billion by mid-2080 [11]. SSA is predicted to have a marked population increase over the next 30 years, reaching 2.2 billion by 2054. Is it estimated that the prevalence of diabetes in the whole of Africa will increase by 142% between 2024 and 2050 [12], which will inevitably adversely influence the healthcare burden of MASLD. In contrast, the populations of Europe and China are predicted to contract, with China’s population forecast to decrease by approximately 150 million over the next 30 years from current estimates of ~1.4 billion [11]. However, in contrast, and based on 2021 Global Burden of Disease (GBD) Study data, the age-standardised prevalence of diabetes among people living in China is projected to increase from 6.2% to 11.1% between 2021 and 2050 [13].

In this review we describe global incidence and prevalence estimates for MASLD, highlighting the variability in epidemiology across different populations, with a focus on the SSA and Asia Pacific regions. We investigate why the known and estimated prevalence and health outcomes of MASLD differ within and across populations, and identify opportunities to advance equity in MASLD prevention, diagnosis and treatment worldwide. We also discuss the impact of social determinants of health on MASLD, including socioeconomic conditions, structural inequities, care access, food environments and systemic discrimination.

## Epidemiology of MASLD

Different approaches have been used to identify hepatic steatosis, which is a key factor in the diagnosis of MASLD. These different approaches are likely to result in different incidence and prevalence estimates, as the sensitivity of different measurement tools to detect liver fat varies. Tools used include ultrasound, MRI, the controlled attenuation parameter, computed tomography and liver biopsy; their performance and comparability have been described previously in a review of the global epidemiology of MASLD [14].

On the basis of the limited available data, it has been estimated that about one-third of the global adult population is living with MASLD, making it the most common chronic liver disease worldwide [14–16]. A 2021 systematic review and meta-analysis of the prevalence and incidence of MASLD identified 72 publications from 17 countries with a total population of 1,030,160 individuals [17]. Data from all 17 countries were included in the prevalence estimates, and data from five countries, with a sample population of 381,765 individuals, were included in the incidence estimates [17]. The estimated overall global prevalence and incidence of MASLD were 32.4% (95% CI 29.9, 34.9; range 20–58) and 46.9 cases per 1000 person-years (95% CI 36.4, 57.5; range 28–60), respectively. However, the value of these summary estimates is questionable given the heterogeneity (illustrated by *I*^2^ values of 99.9% for both prevalence and incidence) of estimates in the individual studies [17]. Among people with type 2 diabetes, a 2023 meta-analysis of data from 156 studies performed in 36 countries (including Nigeria, Sudan and Ethiopia) that included a total of about 1.8 million people reported prevalence estimates for MASLD and MASH of 65% (95% CI 62, 68; range 41–94) and 32% (95% CI 17, 51; range 0.49–96.8), respectively, again with considerable heterogeneity (*I*^2^ values of 90–100%) between studies [18]. An updated search of the literature did not find any more recent population-based studies of MASLD/MASH prevalence among people with type 2 diabetes. These meta-analyses illustrate both the large number of countries for which data are not available, such that extrapolation of data from other countries is required, and the marked heterogeneity of prevalence and incidence estimates across different populations, which is related to differences in approaches to ascertainment and differences in population selection and characteristics.

In contrast to the meta-analyses discussed above, the GBD Study attempts to address cross-regional differences in diseases by mapping disease-specific case definitions to a standardised framework and applying statistical modelling to adjust for variations in diagnostic methods, study design and population characteristics. GBD estimates are derived from multiple data sources, including routine data with ICD codes, population-based surveys, cohort studies and systematic reviews, which are then synthesised and adjusted using the DisMod-MR model to generate internally consistent estimates of incidence, prevalence and mortality from the available data. Original estimates from individual sources and the heterogeneity between them are often not described in publications reporting the pooled estimates. Inevitably, the accuracy and completeness of the information used by the GBD Study reflect variations in data collection and reporting standards across different countries/regions. In addition GBD estimates of MASLD prevalence are not stratified by diabetes status. However, the GBD Study generates global estimates of overall MASLD prevalence using age–period–cohort models and the sociodemographic index to extrapolate available data across all countries that provide initial estimates, which can be improved when further primary data become available.

**Fig. 1:**
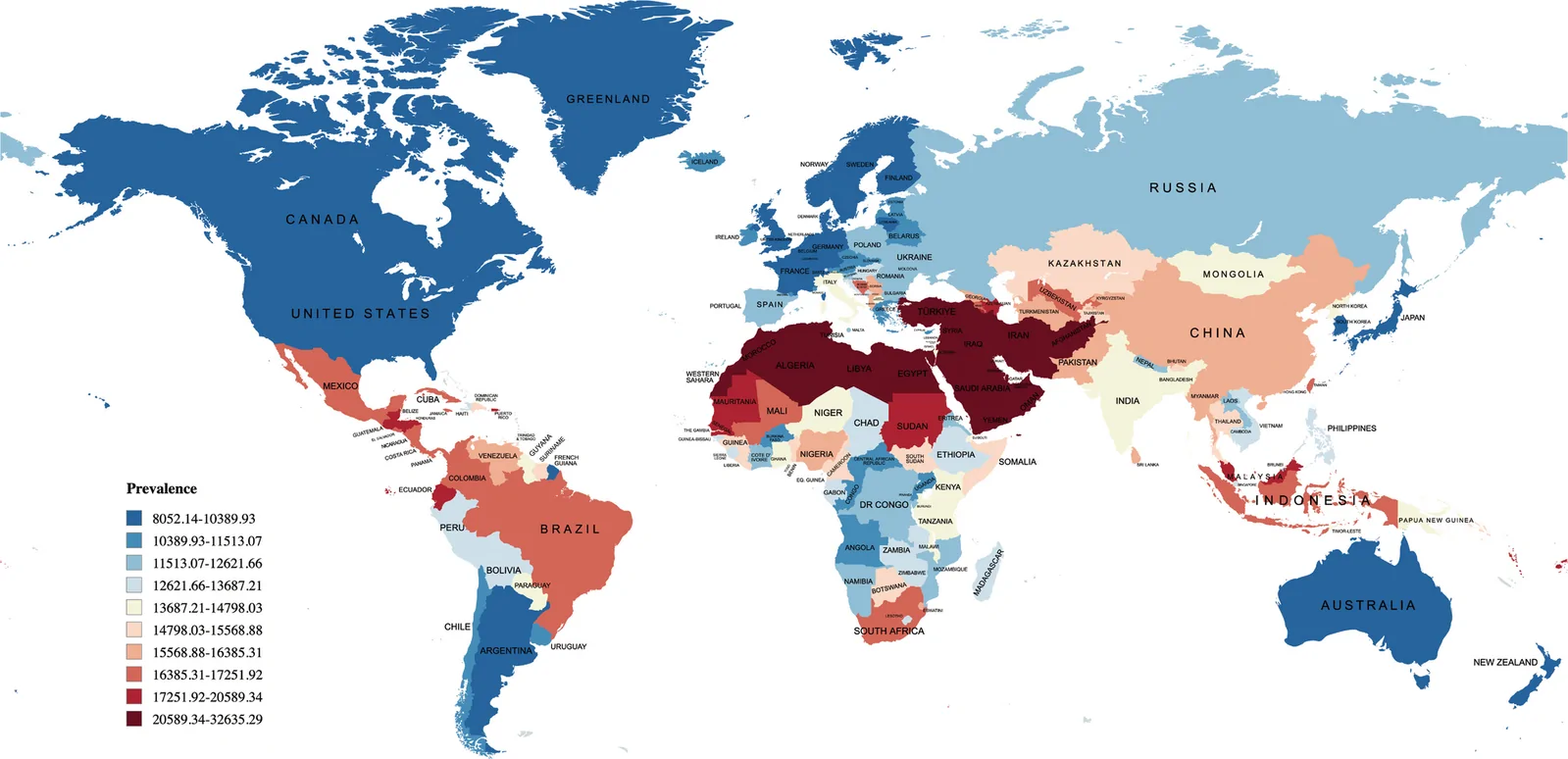
A map of estimates of MASLD prevalence (and 95% UIs) per 100,000 population from the 2021 GBD Study (adapted from [19] with permission). Created with mapchart.net. This figure is available as a downloadable slide

The 2021 GBD Study estimated that the global age-standardised 2021 prevalence and incidence of MASLD were 15,018 per 100,000 population (95% uncertainty index [UI] 13,756–16,361) and 593 per 100,000 population (95% UI 543–644), respectively [19]. A map of estimates by country of MASLD prevalence (and 95% UIs) per 100,000 global population from the 2021 GBD Study is shown in Fig. 1. GBD 2021 estimates of MASLD prevalence and incidence for ten regions used in GBD studies are provided in Table 1 (data for the SSA and Asia Pacific regions are described in subsequent tables) [19]. The highest prevalence estimates were for North Africa and the Middle East and the lowest were for North America, Southern Latin America and Western Europe.

**Table 1:** Age-standardised GBD 2021 estimates of MASLD prevalence and incidence for ten selected level 2 regions as defined in the GBD Study

Region (level 2)^a^	Prevalence per 100,000 population (95% UI)	Incidence per 100,000 population (95% UI)
Andean Latin America	14,985 (13,708–16,362)	611 (583–647)
Caribbean	15,651 (14,340–16,986)	603 (568–639)
Central Europe	12,732 (11,619–13,853)	506 (492–522)
Central Latin America	16,984 (15,537–18,534)	714 (692–735)
Eastern Europe	12,294 (11,255–13,359)	494 (455–544)
North America	10,056 (9187–10,926)	422 (402–444)
North Africa and the Middle East	27,687 (25,587–29,915)	1076 (1045–1104)
Southern Latin America	10,293 (9395–11,265)	408 (378–443)
Tropical Latin America	16,663 (15,245–18,206)	698 (624–798)
Western Europe	10,842 (9939–11,802)	512 (491–534)

Data for the SSA and Asia Pacific regions are provided in Tables 2 and 4, respectively

^a^The GBD regional hierarchical structure is as follows: level 0 – global; level 1 – super-regions (*n*=7); and level 2 – regions (*n*=21). Estimates in this table are selected from level 2 data

Data from [19]

Mortality rates for MASLD appear to be rising globally, with significant increases in liver-related mortality rates reported in some populations and in cardiovascular or non-liver cancer mortality rates among people with MASLD compared with those without. Data on deaths attributed to MASLD in the USA have been reported for adults aged ≥25 years from 1 January 2006 to 31 December 2023 [20]. MASLD-related mortality increased rapidly between 2006 and 2023 and was projected to rise over the next 20 years, with the largest increases in those aged ≥65 years, in non-Hispanic White and Hispanic individuals and in non-metropolitan populations [20]. In order to describe relative all-cause and cause-specific mortality in MASLD, people with ICD-10 codes for MASLD in inpatient or specialised outpatient care in Swedish healthcare registers for 2002–2020 (*n*=13,099) were matched with up to ten control individuals (median, *n*=9) from the general population (*n*=118,884) [21]. MASLD was associated with higher all-cause mortality (HR 1.85, 95% CI 1.74, 1.96) and higher rates of all specific causes of death except mental health disorders. The strongest associations were observed for non-HCC liver-related (HR 26.9, 95% CI 19.4, 37.3) and HCC-related (HR 35.0, 95% CI 17.0, 72.1) mortality. However, the highest estimated 15 year cumulative causes of death in individuals with MASLD were for non-HCC cancer (7.3%) and CVD (7.2%) [21].

### MASLD in the sub-Saharan Africa region

The reported prevalence of MASLD in Africa among the general population is 13.5% (95% CI 5.67, 28.70), but this is likely to be an underestimate because of a scarcity of data related to limited diagnostic capacity, lack of awareness and challenges in healthcare access [17, 22]. According to the 2021 GBD Study, there are marked regional disparities in the estimated prevalence of MASLD within SSA [19]. As reported in Table 2, the highest age-standardised prevalence and incidence estimates in SSA in 2021 were in Southern SSA and the lowest in were in Central SSA. From 2010 to 2021, the largest estimated percentage increases in age-standardised prevalence and incidence occurred in Southern SSA (7.1%, 95% UI 5.6–8.8) and Western SSA (4.3%, 95% UI 2.5–6.1), respectively [19]. Absolute MASLD numbers are increasing in several countries including Nigeria and Ghana (Western SSA), Kenya (Eastern SSA) and South Africa (Southern SSA).

**Table 2:** Age-standardised GBD rates (per 100,000 population) of global and SSA regional MASLD prevalence and incidence in 2021, and percentage changes in prevalence and incidence from 2010 to 2021

Region (level 0 and level 2)^a^	Prevalence			Incidence		
	No. of cases (95% UI)	ASR per 100,000 population (95% UI)	Percentage change in ASR per 100,000 population (95% UI)	No. of cases (95% UI)	ASR per 100,000 population (95% UI)	Percentage change in ASR per 100,000 population (95% UI)
Global	1,267,867,997 (1,157,934,071–1,380,435,423)	15,018.1 (13,756.5–16,381.4)	11.2 (10.5–11.8)	48,353,272 (47,612,534–49,094,010)	608.5 (598.8–617.7)	3.2 (2.1–4.2)
Western SSA	47,962,036 (43,856,187–53,002,936)	14,936.8 (13,659.3–16,347.6)	6.2 (5.3–7.2)	2,501,856 (2,465,846–2,537,866)	511.6 (490.5–534.7)	4.3 (2.5–6.1)
Central SSA	10,850,618 (9,833,003–11,986,283)	11,870.6 (10,844.9–12,943.4)	5.6 (3.1–8.5)	549,883 (541,760–558,005)	397.2 (333.7–486.3)	−0.4 (−4.0 to 3.6)
Eastern SSA	37,304,081 (34,041,055–41,466,666)	13,162.1 (12,037.1–14,400.2)	6.5 (5.5–7.5)	1,953,106 (1,325,028–1,981,184)	455.5 (440.7–471.3)	−0.9 (−2.4 to 0.6)
Southern SSA	11,781,789 (10,763,174 −12,907,446)	15,937.2 (14,572.6–17,388.0)	7.1 (5.6–8.8)	532,714 (524,679–540,749)	666.8 (616.9–722.6)	1.9 (−0.5 to 4.3)

^a^The GBD regional hierarchical structure is as follows: level 0 – global; level 1 – super-regions (*n*=7); and level 2 – regions (*n*=21). Estimates in this table are from level 0 and from level 2 regions within SSA

ASR, age-standardised rate

Data from [19]

MASLD prevalence in SSA is increasing as a result of increasing rates of obesity and type 2 diabetes related to rising urbanisation and associated food insecurity, consumption of energy-dense ultra-processed food and sedentary lifestyles [23]. Many SSA countries have experienced progressive GDP growth, while the UN Food and Agriculture Organization has recorded a steady increase in daily energy intake in Africa as a consequence of the nutritional transition [24].

The estimated prevalence of combined overweight and obesity also varies across the region and is highest in Southern SSA and lowest in Eastern SSA as shown in Table 3 [25]. According to the 2021 GBD forecasting study, between 2021 and 2050 the number of people living with overweight and obesity in SSA is predicted to increase by 254.8% (95% CI 234.4, 269.5) [25], which is likely to have a marked impact on the prevalence of MASLD.

**Table 3:** Age-standardised GBD estimates of overweight and obesity prevalence (%) for the world and SSA regions in 1990 and 2021, and percentage changes from 1990 to 2021, among adults aged ≥25 years by sex

Region (level 0 and level 2)^a^	Women			Men		
	Prevalence (95% UI, %)		Relative change in prevalence (95% UI, %)	Prevalence (95% UI, %)		Relative change in prevalence (95% UI, %)
	1990	2021	1990–2021	1990	2021	1990–2021
Global	30.5 (30.2–30.8)	46.7 (46.2–47.2)	53.0 (50.5–55.3)	27.6 (27.4–27.9)	43.4 (42.9–43.8)	56.9 (54.7–59.2)
Western SSA	30.6 (29.0–32.2)	45.2 (43.6–46.9)	47.9 (38.2–57.9)	21.1 (19.7–22.5)	38.7 (37.1–40.3)	83.3 (69.3–97.9)
Central SSA	18.1 (16.5–19.7)	36.2 (33.8–38.6)	100.5 (79.5–124.4)	12.7 (11.6–13.8)	28.8 (27.0–30.9)	128.2 (103.5–154.5)
Eastern SSA	19.7 (18.8–20.6)	31.7 (30.8–32.6)	61.2 (52.3–70.3)	11.4 (10.9–11.9)	23.3 (22.4–24.2)	104.5 (93.2–116.8)
Southern SSA	53.7 (51.5–55.9)	71.1 (69.3–72.9)	32.5 (26.4–38.5)	28.4 (26.9–30.3)	45.5 (43.4–47.7)	60.3 (48.3–73.1)

^a^The GBD regional hierarchical structure is as follows: level 0 – global; level 1 – super-regions (*n*=7); and level 2 – regions (*n*=21). Estimates in this table are from level 0 and from level 2 regions within SSA

Adapted from [25]

Age-standardised 2021 GBD estimates of diabetes prevalence per 100,000 population also vary by region at 5602.3 (95% UI 5225.5–6018.8), 5263.8 (95% UI 4847.9–5638.8), 4678.0 (95% UI 4348.9–4982.2) and 2921.9 (95% UI 2716.9–3097.0) in Southern, Central, Western and Eastern SSA, respectively [13]*.* Concerningly, as mentioned above, the IDF predicts that Africa will experience the largest relative increase in diabetes prevalence globally between 2024 and 2050 [12].

A meta-analysis of data from SSA found that 41.8% (95% CI 33.2, 50.8; *I*^2^=99.5%) of people living with food insecurity had one or more MASLD-related metabolic risk factors. The most prevalent risk factors were dyslipidaemia (27.6%; 95% CI 6.5, 54.9), hypertension (24.7%; 95% CI 15.6, 35.1) and overweight (15.8%; 95% CI 10.6, 21.7) [26].

The MASLD liver-related mortality rate in SSA is estimated to be 2.34 per 100,000 population (95% CI 1.16, 5.15) and the clinical impact of MASLD is further compounded by the coexisting burden of chronic viral hepatitis and alcohol-related liver disease [19, 27, 28]. Genetic variation in key genes also plays a role in MASLD development, but there is a paucity of genetic data relevant to MASLD from SSA. Because of the limited access to MASH-directed pharmaceuticals such as resmetirom and semaglutide, as a result of cost and limited availability, management of MASLD in SSA is dependent on lifestyle changes focused on weight management, improved dietary balance and regular physical activity, along with the avoidance of smoking and alcohol use, and the aggressive and cost-effective management of cardiometabolic risk factors [29]. A public health approach will be particularly important in SSA to address the growing burden of MASLD. For example, health policies should focus on improving access to high-quality food to enable reductions in unhealthy food consumption, promoting physical activity and enhancing healthcare access.

### MASLD in the Asia Pacific region

It has been estimated that MASLD is the leading cause of chronic liver disease in the Asia Pacific region, with a prevalence of around 30% among adults [30]. MASLD prevalence appears to have increased by over 50% in the past two decades, and more than half of estimated global MASLD cases are now found in this region, where approximately 60% of the world’s population lives [31]. The 2021 GBD Study estimates [19, 32] of MASLD prevalence and incidence are shown in Table 4 for the following Asia Pacific sub-regions: Central Asia, South Asia, Australasia, high-income Asia Pacific, East Asia, Southeast Asia and Oceania [19, 32]. Countries within Oceania had the highest estimated prevalence, with Fiji (20,184 per 100,000 population; 95% UI 18,413–22,050) and Samoa (16,551 per 100,000 population; 95% UI 15,129–18,058) having the highest prevalence estimates in this region. Malaysia (19,514 per 100,000 population; 95% UI 17,932–21,267) and Indonesia (17,252 per 100,000 population; 95% UI 15,423–18,820) had the highest estimated prevalences within Southeast Asia, and China had the highest rate within east Asia, at 15,607 per 100,000 population (95% UI 14,272–17,017). High-income Asia Pacific regions, including Japan (8133 per 100,000 population; 95% UI 7270–9030) and the Republic of Korea (10,050 per 100,000 population; 95% UI 9201–10,956), had relatively low prevalence estimates. Australasia had intermediate prevalence estimates, with that for Australia being 9405 per 100,000 population (95% UI 8596–10,303).

**Table 4:** Age-standardised GBD rates (per 100,000) of Asia Pacific regional MASLD prevalence and incidence in 2021, and percentage changes in prevalence and incidence from 2010 to 2021

Region (level 2)^a^	Prevalence			Incidence		
	No. of cases (95% UI)	ASR per 100,000 population (95% UI)	Percentage change in ASR per 100,000 population (95% UI)	No. of cases (95% UI)	ASR per 100,000 population (95% UI)	Percentage change in ASR per 100,000 population (95% UI)
Central Asia	15,204,171 (13,885,517–16,673,758)	16,120.10 (14,735.3–17,604.5)	7.1 (5.6–8.6)	583,559 (574,644–592,474)	612.4 (563.3–673.7)	−6.1 (−9.3 to −2.7)
Australasia	3,778,619 (3,457,855−4,124,388)	9468.20 (8665.5–10,349.4)	5.8 (3.2–8.7)	117,958 (116,015–119,902)	383.2 (368.0–400.4)	5.4 (1.7–9.2)
High-income Asia	24,694,242 (22,636,602−26,784,254)	8885.70 (8148.4–9666.7)	6.4 (3.5–8.9)	728,246 (715,952–740,540)	381.9 (367.1–397.2)	5.8 (3.5–8.1)
South Asia	249,790,702 (227,865,117−273,237,523)	14,158.30 (12,940.9–15,445.1)	12.0 (10.9–12.9)	10,765,351 (10,602,018–10,928,683)	563.3 (518.2–614.2)	1.3 (−0.5 to 3.4)
East Asia	301,408,386 (274,406,342−328,824,040)	15,596.20 (14,262.4–16,999.3)	16.6 (14.5–18.5)	9,905,423 (9,743,925–10,066,921)	660.4 (625.4–699.0)	10.7 (9.1–12.6)
Oceania	1,625,693 (1,483,817−1,796,525)	15,182.70 (13,936.2–16,584.7)	2.1 (–0.0 to 4.4)	76,654 (75,506–77,802)	549.0 (507.1–603.1)	−5.8 (−9.5 to −1.6)
Southeast Asia	115,103,788 (104,841,848−125,940,613)	15,691.70 (14,308.3–17,127.2)	5.7 (4.6–6.7)	4,606,197 (4,535,042–4,677,353)	651.9 (635.1–669.3)	−0.7 (−2.1 to 0.6)

^a^The GBD regional hierarchical structure is as follows: level 0 – global; level 1 – super-regions (*n*=7); and level 2 – regions (*n*=21). Estimates in this table are from level 2 regions within Asia Pacific

ASR, age-standardised rate

Data from [19]

## Social determinants of health relevant to MASLD

The rising prevalence of MASLD across the globe is deeply intertwined with social and economic factors [33, 34]. Urbanisation, rapid lifestyle shifts and socioeconomic disparities contribute to the disease’s growing burden. Changes in the food environment, discussed further below, combined with sedentary lifestyles/limited physical activity and resulting increases in obesity all contribute [34, 35]. Job stress, long working hours and physical labour further exacerbate the risk of metabolic diseases, including MASLD [36–38]. Limited healthcare access contributes to under-diagnosis of MASLD and this reduces the opportunity to prevent severe liver complications.

Despite the high prevalence of MASLD, awareness of MASLD remains low. Approximately 25% of individuals with MASLD are unaware of their condition, which underscores both the low public awareness and the lack of systematic screening strategies [39]. This knowledge deficiency is particularly evident among healthcare providers (HCPs). For example, in Australia, only around 40% of primary care physicians can accurately identify high-risk MASLD patients [40]. In Southeast Asia, 79.1% of HCPs report inadequate training in obesity management, lifestyle counselling and patient communication [41]. These gaps in training and awareness contribute to delayed diagnosis, mismanagement of the disease and missed opportunities for early intervention. Moreover, social and cultural factors, including language barriers and stigma surrounding obesity, can further exacerbate the challenge of addressing MASLD, particularly among marginalised communities. As a result, people are less likely to seek timely care or engage in preventive health measures, further perpetuating the cycle of health inequity.

### Structural inequities

Structural inequities are a central determinant in the increasing burden of MASLD [42]. These inequities are deeply embedded in socioeconomic and healthcare systems and are exacerbated by rapid urbanisation and social stratification [43, 44]. As rural populations migrate to urban centres, informal settlements or urban slums emerge, characterised by overcrowding, poor sanitation and limited access to healthcare. Just as food insecurity is relevant to many common chronic non-communicable diseases (NCDs), it is also very relevant to MASLD. Poor nutrition, marked by a shift towards energy-dense processed foods and a decline in traditional, healthier diets, combined with increasingly sedentary lifestyles, creates an environment conducive to the development of not only obesity and type 2 diabetes, but also MASLD. Socioeconomic status and ethnic disparities further compound the issue, with marginalised populations often facing systemic barriers to accessing healthcare [45]. In countries with rigid social hierarchies, lower income groups and minority ethnic groups are often concentrated in areas with poor healthcare infrastructure. As a result, early detection of MASLD is frequently delayed, and these populations often present with more advanced stages of liver disease [46]. Additionally, cultural and linguistic barriers further hinder access to preventive care and health education, reinforcing the cycle of health inequity.

Policy responses to MASLD are varied. For example, within the Asia Pacific region, high human development index (HDI) countries such as Australia, Japan and South Korea implement more comprehensive strategies, including public health campaigns, physical activity guidelines and childhood obesity prevention measures [47, 48]. In contrast, medium-to-high HDI countries such as China, India and Indonesia are in the process of addressing the rise in metabolic diseases, but these countries face challenges such as outdated health policies, limited public health infrastructure and inadequate screening programmes [49]. For instance to promote improvements in public health, China has introduced initiatives such as the Weight Management Year programme aimed at reducing obesity, while India has revised obesity thresholds in its population to reflect increased risk of type 2 diabetes, compared with people of European ancestry.

### Food environments

There is increasing emphasis on dietary reform, including reducing the intake of processed foods and sugary drinks, using measures such as sugar taxes and restrictions on food advertising, actions that will be crucial to effectively address obesity and MASLD [50]. Food environments have changed rapidly in recent decades, influenced by urbanisation, globalisation and changing cultural trends [51–53]. Across Asia, energy intake has been increasing as a result of increases in dietary sugar and fat consumption [54–57]. Added sugar intake among 3- to 17-year-olds increased more than sevenfold between 1997 and 2011 in Southeast Asia, with many snack foods marketed for children containing added sugars [58]. In Indonesia, the percentage of energy from fat rose from 10% in 1983 to 28% by 2004 [57] and, in parts of Asia, the rise of milk tea culture has become a significant cultural trend, especially among younger people. Milk tea is rich in both added sugar and fat and is no longer just a drink, but a symbol of status and a part of daily social life, especially in countries such as Taiwan and Hong Kong and in parts of mainland China. The availability of fresh produce and nutrient-dense options is limited, while cheap, high-energy foods dominate, which contributes to food inequity [59].

### Inequities in access to MASLD care

Healthcare access inequity continues to be a pervasive problem worldwide [43, 60, 61]. Inequity in healthcare access is partly driven by a lack of awareness about disease, which is common with MASLD. Despite being the most prevalent liver disease, MASLD remains largely unknown and thus unaddressed globally [15].

In a global survey of HCPs, lack of awareness was the most common reason that physicians provided for not screening people for MASLD [62]. Another study found that <3% of people living with MASLD were aware of their diagnosis [63]. This dearth of MASLD awareness among those living with the disease and the HCPs responsible for treating it, leads to a suboptimal distribution of healthcare resources. If people have no knowledge of their disease risk, they will be unlikely to seek care, which together with HCPs’ lack of disease awareness, results in an absence of detection and more people living with the disease who are undiagnosed (and untreated).

Public health strategies targeting MASLD are limited. At a global level, a study of 102 countries found that no country had a comprehensive MASLD public health response, as exemplified by the dearth of strategic documentation across all nations [64]. Moreover, MASLD remains unaddressed in the WHO’s guidance, action plan and strategy documents, with the WHO being responsible for setting the tone and direction of health policy worldwide [65]. This absence of a MASLD focus in strategic health planning also affects healthcare resource allocation. Without proper disease recognition via documentation that guides action, MASLD will continue to be overlooked and underfunded. This will ultimately also result in people continuing to live at risk of potentially preventable liver disease progression.

In September 2025, the UN included MASLD in the political declaration of its fourth high-level meeting on NCDs [65], although the disease was still not recognised as a high-priority NCD. Thus, although this represents progress in the right direction, the UN and WHO could go further and place MASLD at the core of the global NCD agenda. Only by giving MASLD such recognition will it be possible to reverse the global healthcare burden of MASLD as a multisystem disease that has disease ramifications beyond the liver. Without recognising MASLD as a high-priority NCD, MASLD will remain a public health threat that undermines the global pledge to reduce NCD-related mortality by 2030 [66].

Access to care for MASLD is a significant challenge across much of the Asia Pacific and SSA region, especially in rural and underserved areas. One of the primary barriers is the lack of diagnostic tools. As a result, many individuals remain unaware of their condition until it has progressed significantly. Geographical barriers further complicate access, with healthcare services often concentrated in urban centres. In Asia Pacific countries such as India, Indonesia and Myanmar and in many countries in Africa, rural populations must travel long distances to reach even basic medical facilities, incurring high costs and facing limited healthcare resources. Moreover, primary care physicians in these regions often lack training in liver disease diagnosis and MASLD risk assessment, which exacerbates the issue of delayed detection. In addition, methods for identifying liver fibrosis, the important predictor of major adverse liver outcomes, may not be available, particularly in low- and middle-income countries.

Pharmacological treatments for MASH, including semaglutide, the glucagon-like peptide-1 receptor agonist (GLP-1RA), and resmetirom [67], a selective thyroid hormone receptor-beta (THR-β) agonist, were licensed for the treatment of F2 and F3 liver fibrosis in some high-income countries in 2024 and 2025. Semaglutide, in particular, has widespread benefits beyond the liver in reducing the risk of CVD and chronic kidney disease and treating obesity and hyperglycaemia in type 2 diabetes. However, in addition to limited availability, the cost of widespread use of these drugs is often prohibitive for health systems, and access to new therapies is often delayed. Table 5 summarises the actions and benefits of currently licensed key pharmacological treatments known to benefit liver disease, obesity, type 2 diabetes and CVD in MASLD, as part of a multisystem disease. It should be noted that Table 5 is not meant to be fully comprehensive, and established treatments and potential new treatments for MASLD are considered in detail in the review by Stefan and Targher [68] in this special issue.

**Table 5:** Summary of licensed pharmacological treatments known to improve outcomes for MASLD as a multisystem disease, many of which have limited availability globally^a^

Characteristic	GLP-1RAs (e.g. semaglutide)	THR-β agonists (e.g. resmetirom)	SGLT2 inhibitors (e.g. dapagliflozin, empagliflozin)	Statins
Key tissue activity	Brain, stomach, pancreas (decrease appetite, slow gastric emptying and increase insulin secretion)	Liver (THR-β agonists)	Kidney (reduce renal glucose reabsorption)	Liver (HMG-CoA reductase inhibitors)
Liver-specific tissue benefit/effect	Reduction in liver fat, inflammation and fibrosis	Reduction in liver fat, inflammation and fibrosis	Uncertain, recent evidence of reductions in liver fat, inflammation and fibrosis Decrease risk of MALO	Uncertain, but probably neutral for effects on liver fibrosis. Safe in MASLD although should be stopped if ALT levels increase
Key benefits	Weight loss Improved glucose tolerance Decreased risk of CKD and MACE Reduction in liver fibrosis	Reduction in liver fibrosis Reduction in cardiovascular risk factors (e.g. LDL-cholesterol, apolipoprotein B-100 and Lp(a)) but uncertain disease benefits beyond the liver	Reduced risk of CKD	Reductions in LDL-cholesterol and risk of MACE
Key side affects	Gastrointestinal side effects (nausea, diarrhoea, constipation)	Gastrointestinal side effects (nausea, diarrhoea, constipation)	Increased risk of genitourinary infection	Increased liver enzymes, muscle aches and, very rarely, rhabdomyolysis

^a^To identify individuals with MASLD for treatment, the 2023 American Association for the Study of Liver Diseases (AASLD) guidance recommends screening for MASLD in high-risk populations, including individuals with type 2 diabetes, obesity with associated metabolic comorbidities, a family history of cirrhosis or significant alcohol use [84]. The 2024 European Association for the Study of the Liver (EASL) clinical practice guidelines strongly recommend screening for MASLD with liver fibrosis in individuals with type 2 diabetes, abdominal obesity plus additional metabolic risk factors, or abnormal liver function tests [85]. In 2025, the ADA acknowledged the clinical importance of MASLD in diabetes management, recommending the use of the FIB-4 index to screen adults with type 2 diabetes or prediabetes, especially those with obesity or other cardiometabolic risk factors, for the risk of developing MASH and liver fibrosis, even if serum liver enzyme levels are normal [86]

CKD, chronic kidney disease; HMG-CoA, 3-hydroxy-3-methylglutaryl CoA; MACE, major adverse cardiovascular events; MALO, major adverse liver outcomes; SGLT2, sodium–glucose cotransporter 2

Geographical isolation, healthcare workforce shortages and financial barriers further restrict access to both diagnostic and therapeutic services. Addressing these barriers would require additional funding and enhancement of primary care training, integration of MASLD diagnosis into routine metabolic health assessments and expansion of telemedicine and mobile health solutions. Additionally, policy initiatives aimed at subsidising diagnostic tests and treatments could help reduce financial barriers and improve access to care. Lifestyle modification, including dietary change, physical activity and weight management, remains the cornerstone of therapy for most patients [69, 70].

### Systemic discrimination

Systemic discrimination exacerbates health inequities in the risk of common NCDs such as obesity, type 2 diabetes and MASLD, disproportionately affecting indigenous populations, minority ethnic groups and migrant communities. For example, in Australia, Indigenous people face higher rates of chronic diseases, including MASLD, partly because of the historical impacts of colonisation and geographical isolation, which limit healthcare access and health literacy [71]. Similarly, minority ethnic groups in all countries often experience discrimination in healthcare settings, leading to poor access to care and delayed diagnoses. Migrant communities in urban areas face economic marginalisation, cultural dislocation and healthcare access barriers, further exacerbating the risk of MASLD [72]. These populations often live in areas with inadequate healthcare infrastructure, making early diagnosis and intervention difficult [72, 73]. Cultural and language barriers add to the challenge, preventing these communities from effectively engaging with healthcare systems and seeking timely care. The politics of attempting to mitigate the interaction between global capitalism and human nature, for example with sugar taxes, is often contentious and further complicates access to inexpensive nutritious options, thus deepening health disparities [74]. To address these systemic issues, targeted policy interventions are needed to reduce discrimination, improve healthcare access and ensure equitable MASLD prevention and treatment for all populations [75].

## Genetic and phenotypic susceptibility

There is a paucity of genetic data available for MASLD, particularly in SSA. However, increasing evidence suggests that genetic factors play an important role in the development and progression of MASLD in the Asia Pacific region. For example, the *PNPLA3* (patatin-like phospholipase domain-containing protein 3) rs738409 G allele (I148M) variant and the *TM6SF2* (transmembrane 6 superfamily member 2) rs58542926 T allele (E167K) variant are both associated with accelerated accumulation of liver fat and rapid fibrosis progression and are more prevalent in Asian populations than in Western populations [76, 77]. Asian individuals with these genetic variants often develop liver damage at a lower BMI than non-Asian individuals, suggesting that those who do not meet traditional obesity criteria may still be at risk for advanced liver disease [78]. Many Asian individuals accumulate visceral fat at a lower BMI (than non-Asian individuals), leading to the phenomenon of ‘lean MASLD’ [79]. This condition, where individuals with a BMI of <23 kg/m^2^ experience significant liver fat deposition and metabolic dysfunction, accounts for 10.8–19.3% of MASLD cases in Asia [80–82]. Despite a lower BMI, these individuals face disease progression similar to that in individuals with obesity, highlighting the critical role of visceral fat and ectopic lipid deposition. The genetics of MASLD is considered in more detail in the review by Bilson and Yaghootkar [83] in this special issue.

## Conclusion

MASLD is a multisystem disease with ramifications beyond the liver for commonly comorbid NCDs such as type 2 diabetes and CVD. The limited available evidence suggests that the global burden of MASLD is high and increasing, not just in SSA and the Asia Pacific regions (on which this review has focused), but also in the rest of the world. Obesity and type 2 diabetes are important risk factors that are having a major impact on MASLD incidence and prevalence across the globe. The presence of type 2 diabetes increases the risk of liver fibrosis, cirrhosis and HCC in MASLD and, in people who have developed MASLD (but who do not have type 2 diabetes), MASLD is an independent risk factor for developing incident type 2 diabetes. Thus, type 2 diabetes and MASLD form part of a vicious spiral of worsening disease outcomes, with type 2 diabetes influencing liver disease and liver disease influencing diabetes development [6–8]. Public health strategies targeting obesity, the food environment, food insecurity and physical activity are key to ameliorating the healthcare burden of MASLD, even in countries where pharmacological treatments are available. MASLD should be a central part of the global NCD agenda. Better coverage and quality of prevalence and incidence data would support resource allocation, facilitating better planning and evaluation of health services focused on MASLD. Initiatives that recognise MASLD as a multisystem disease with consequences for other organ systems beyond the liver are urgently required. Only then may it be possible to achieve the commitment made by countries to reduce NCD-related mortality by 2030.

## Supplementary Information

Below is the link to the electronic supplementary material.

Figure slide (PPTX 932 KB)

## Abbreviations

GBD: Global Burden of Disease
GLP-1RA: Glucagon-like peptide-1 receptor agonist
HCC: Hepatocellular carcinoma
HCP: Healthcare provider
MASH: Metabolic dysfunction-associated steatohepatitis
MASLD: Metabolic dysfunction-associated steatotic liver disease
NAFLD: Non-alcoholic fatty liver disease
NCD: Non-communicable disease
SSA: Sub-Saharan Africa
THR-β: Thyroid hormone receptor-beta
UI: Uncertainty index
UN: United Nations
